# NOTIFy (non-toxic lyophilized field)-FISH for the identification of biological agents by Fluorescence *in situ* Hybridization

**DOI:** 10.1371/journal.pone.0230057

**Published:** 2020-03-06

**Authors:** Karin Aistleitner, Tina Sieper, Inga Stürz, Rimma Jeske, Susanne Tritscheller, Sonja Mantel, Alina Tscherne, Sabine Zange, Kilian Stoecker, Roman Wölfel

**Affiliations:** Bundeswehr Institute of Microbiology, Munich, Germany; University of Salento, ITALY

## Abstract

The rapid and reliable diagnostics of highly pathogenic bacteria under restricted field conditions poses one of the major challenges to medical biodefense, especially since false positive or false negative reports might have far-reaching consequences. Fluorescence in situ hybridization (FISH) has the potential to represent a powerful microscopy-based addition to the existing molecular-based diagnostic toolbox. In this study, we developed a set of FISH-probes for the fast, matrix independent and simultaneous detection of thirteen highly pathogenic bacteria in different environmental and clinical sample matrices. Furthermore, we substituted formamide, a routinely used chemical that is toxic and volatile, by non-toxic urea. This will facilitate the application of FISH under resource limited field laboratory conditions. We demonstrate that hybridizations performed with urea show the same specificity and comparable signal intensities for the FISH-probes used in this study. To further simplify the use of FISH in the field, we lyophilized the reagents needed for FISH. The signal intensities obtained with these lyophilized reagents are comparable to freshly prepared reagents even after storage for a month at room temperature. Finally, we show that by the use of non-toxic lyophilized field (NOTIFy)-FISH, specific detection of microorganisms with simple and easily transportable equipment is possible in the field.

## Introduction

Certain highly pathogenic bacteria like *Bacillus anthracis*, *Yersinia pestis* or *Burkholderia mallei* do not only cause life-threatening disease in humans by naturally occurring infections, but they can and have been deliberately used as biological agents in terrorist attacks or offensive biological warfare programs [1]. This potential has led to the development of numerous nucleic acid- and antigen-based assays for detection and identification of these organisms over the past years (e.g. [2–5]). Such assays allow high-throughput analyses and represent the diagnostic gold-standard regarding sensitivity. However, they suffer from the immanent problem that they cannot discriminate between active and dead organism or the contamination of a sample with genomic or plasmid DNA. In addition, they are prone to inhibition by diverse sample matrices and their application in the laboratory poses great demands in terms of logistics, laboratory equipment and staff training. In order to mitigate these challenges, Fluorescence in situ Hybridization (FISH) represents a valuable method to complement the existing molecular biology-based toolbox for the diagnostics of highly pathogenic bacteria. FISH is a microscopy-based method for the detection of ribosomal RNA by fluorescently labeled oligonucleotide probes. It is widely used for the identification of microorganisms in microbial ecology [6], but also for the identification of pathogens in food microbiology and in clinical settings [7–12]. Since the detection of an organism by FISH requires intact cells with at least 400 ribosomal target molecules per cell [13], lysed or dead cells are not detected by this method. Because of this, FISH is a valuable complement to nucleic acid- or antigen-based methods despite its lower sensitivity (e.g. [14, 15]). Furthermore, novel probe-labeling strategies have been developed and employed for FISH in the past years. This has resulted in increased signal intensities, the differentiation of more bacterial populations with a single hybridization, and faster turnaround times: For example, the Double-labeling of Oligonucleotide ProbEs (DOPE-FISH) methods allows for the detection of up to six different populations in a single experiment [16]. DOPE-FISH additionally results in higher signal intensities due to the incorporation of more than one fluorophore per bound probe [13, 16]. The more advanced CLASI-FISH further increases the possible number of target organisms in a single hybridization by Combinatorial Labeling And Spectral Imaging [17]. In clinical settings, FISH-probe sets targeting a set of bacteria have been used for the identification of bacteria in respiratory disease, bloodstream infections, chorioamnionitis or endocarditis (e.g. [18–22]). However, these studies all relied on parallel or subsequent hybridizations with a maximum of three probes per reaction in order to identify the relevant bacteria. The causative agents of anthrax, plague, tularemia and brucellosis have already been targeted by FISH in previous studies [23–28], but a comprehensive probe set for multiplexed in situ detection of a wide range of biodefense relevant bacteria has hitherto not been established. The situation is further complicated by the fact that first preliminary tests for biological agents might have to be performed on site in deployed field laboratories. Under such resource limited conditions the need for fast and easy transport limits the size and weight of equipment [29]. Moreover, hazardous chemicals should be avoided if possible, as they pose challenges for air transport as well as for handling in a deployed field laboratory: FISH is routinely performed in the presence of formamide, a denaturing agent that reduces the thermal stability of the double-stranded probe-target-complex, increases the accessibility to the rRNA target and competes for hydrogen bonding [30]. Adjustment of the formamide concentration for each probe ensures stringent binding of the probe to the target organism at the chosen hybridization temperature. However, its volatile and hazardous nature at the temperature used for hybridizations makes formamide unsuitable for use in the field. First attempts to replace formamide by other chemicals have been published [31–33], but have not found broad application so far.

In this study, we developed a set of FISH-probes to identify bacterial biological agents and related pathogens of high clinical relevance for differential diagnosis. To allow for easy application in a field laboratory, and, even more important, cold chain independent transport of all required components, we optimized the conventional FISH protocol. The substitution of formamide with non-toxic urea, followed by the lyophilization of all reagents in combination with the corresponding easily transportable equipment [29], allows this approach to complement nucleic acid amplification- and antigen-based methods in field laboratories. In addition, we established a workflow for the identification of thirteen organisms within four hours and applied it to different sample matrices relevant for bio-terroristic agents as well as clinical samples.

## Materials and methods

### Cultivation and fixation of organisms

Bacterial strains, growth media and temperatures used in this study are listed in Table 1 for target organisms and S1 Table for organisms serving as non-target controls for the evaluation of FISH-probes. Bacteria classified as biological risk group 3 organisms were grown, handled and fixed in a BSL-3 laboratory using appropriate personal protective equipment. To prepare samples for FISH, pure cultures were pelleted by centrifugation and washed once in phosphate buffered saline (PBS). Cells were resuspended in PBS, three volumes of 4% formaldehyde were added and cells were fixed for two hours at room temperature. *Bacillus anthracis* was fixed with 10% formaldehyde to ensure inactivation of spores. For liquid clinical samples, three volumes of 4% formaldehyde were added to the sample, followed by fixation for two hours at room temperature. Swabs were placed in a 15 ml falcon with 3 ml PBS, vortexed and fixed with formaldehyde as described above. Following fixation, bacteria were washed three times in PBS, resuspended in a 1:1 mixture of PBS and ethanol and stored at -20°C. The obligate intracellular bacteria *Rickettsia slovaca* and *Coxiella burnetii* were grown in Vero E6 cells and Buffalo green monkey cells in GlutaMAX minimal essential medium (GIBCO) supplied with 1% non-essential amino acids (GIBCO) and 5% fetal bovine serum or minimal essential medium with 1% non-essential amino acids and 2 mmol L-glutamine, respectively. Host cells containing *R*. *slovaca* were scraped from cell culture flasks, pelleted by centrifugation and lysed by five passages through 26 gauge needles. Host cell debris was removed by centrifugation for 5 minutes at 117 x g, the remaining supernatant was centrifuged at 11,363 x g for 10 minutes to pellet rickettsiae and the bacteria were fixed as described above. For *C*. *burnetii*, cell culture supernatant containing released bacteria was fixed as described above. All clinical samples for molecular diagnostics were leftover material from diagnosing of patients, which would have been discarded otherwise. No extra sampling from the patients was performed. The work with clinical samples has been carried out in-line with “The Code of Ethics of the World Medical Association (Declaration of Helsinki)” and according to good clinical practice guidelines. According to the local legislation, no formal approval of a research ethics committee was needed, because there were anonymized and for research purposes stored samples used.

**Table 1:** Organisms and growth conditions used in this study.

Organism	Strain	Growth temperature	Medium
*Bacillus anthracis*	CDC 1014	37°C	LB (DSMZ 381)
*Brucella melitensis*	ATCC 23456	37°C	Columbia blood agar (DSMZ 693)
*Burkholderia pseudomallei*	ATCC 15682	37°C	LB (DSMZ 381)
*Burkholderia mallei*	ATCC 15310	37°C	Columbia blood agar (DSMZ 693)
*Coxiella burnetii*	9 mile	37°C	Buffalo green monkey cells (European Collection of Cell Cultures, Salisbury, U.K)
*Escherichia coli*	Nissle	37°C	LB (DSMZ 381)
*Francisella tularensis holarctica*	ATCC 29684	37°C	Brain heart infusion (DSMZ 215)
*Leptospira borgpetersenii*		37°C	Difco Leptospira medium (DSMZ 1113)
*Rickettsia slovaca*	Rom 828	30°C	Vero E6 cells (ATCC CRL-1586)
*Vibrio cholerae*	2470–80	37°C	LB (DSMZ 381)
*Yersinia pestis*	EV 76	28°C	LB (DSMZ 381)
*Yersinia pestis*	KIM pgm+	28°C	LB (DSMZ 381)
*Yersinia pestis*	Yokahama	28°C	LB (DSMZ 381)
*Yersinia pestis*	M23	28°C	LB (DSMZ 381)
*Yersinia pestis*	TS pgm+	28°C	LB (DSMZ 381)
*Staphylococcus aureus*	ATCC 29213	37°C	LB (DSMZ 381)
*Neisseria meningitidis*	DSMZ 10036	37°C	LB (DSMZ 381)
*Neisseria gonorrhoeae*	DSM-9188	37°C	LB (DSMZ 381)

### Probe design and validation for FISH

Probes were designed using the arb 6.0 software package [34] and the SILVA SSU and LSU databases [35] and were subsequently further checked by BLAST for binding to non-target organisms. Probes labeled with either one or two molecules of 5(6)-carboxyfluorescein-N-hydroxysuccinimide ester (FLUOS), indocarbocyanine (Cy3) or indodicarbocyanine (Cy5) (all: Metabion, Munich, Germany). Probes with locked nucleic acids (LNA) were synthesized by TIB Molbiol (Berlin, Germany). For each newly designed probe, the optimal hybridization condition was determined by formamide series as previously described [36]. The respective non-target organism to test the specificity of new probes in these experiments was chosen based on the SILVA database as the microorganism containing the lowest number of mismatches to the probe. For some probes unlabeled competitor or helper probes were designed and included in the hybridizations to improve the specificity or signal intensity of the respective probe [37, 38]. Probes used in this study are listed in Table 2.

**Table 2:** FISH-probes used in this study.

Probe	Specificity	Target molecule	Sequence (5'-3')	Coverage^§^	Non target hits^§^	Ref
Bac1157*	*B*. *anthracis*/*cereus* group	23S rRNA	CCA TTG GTA TCA ATC CGC AGC	99.4%	0	
Bboth16S454*	*B*. *mallei* and *B*. *pseudomallei*	16S rRNA	CAC TCC GGG TAT TAG CCA GAA TG			[39]
Bet42a	most *Betaproteobacteria*	23S rRNA	GCC TTC CCA CTT CGT TT			[37]
Bmal16S95*	*B*. *mallei*	16S rRNA	CGT TCA CCA CTC GCC A			[39]
Bru-996*	*Brucella* spp.	16S rRNA	CCA CTA ACC GCG ACC GGG ATG			[26]
Bwall1448*	*F*. *tularensis*	23S rRNA	CAA CCA TTC GCC AGG CCT			[25]
Coxb0187	*C*. *burnetii*	16S rRNA	ATC CCC CGC TTT GCT CCA A			[40]
Esco864*	*E*. *coli*/*Shigella*	23S rRNA	CCC TTG CCG AAA CAG TGC	99.7%	0	
Gam42a	most *Gammaproteobacteria*	23S rRNA	GCC TTC CCA CAT CGT TT			[37]
LGC354B	Firmicutes	16S rRNA	CGG AAG ATT CCC TAC TGC			[41]
Lep0673	*Leptospira* spp.	23S rRNA	GGA TAG CTC GAC AGG CTT CG	99.1%	0	
NeMe183*	*N*. *meningitidis*	16S rRNA	CCT GCT TTC TCT CTC AAG A			[15]
Rick2287	*Rickettsia* spp.	23S rRNA	CCA ACC TGA GCT AAC CAT CG	99.3%	0	
Sau*	*S*. *aureus*	16S rRNA	GAA GCA AGC TTC TCG TCC G			[42]
Vchol1405*	*V*. *cholerae*	23S rRNA	CCC CAT CGC AAT AGT CAG AA	99.5%	0	
Ypest1531LNA*	*Y*. *pestis*	23S rRNA	CTG CTT CTG CAC CGT +G GTG	100%	0	
**Control probes**						
EUB338	most Bacteria	16S rRNA	GCT GCC TCC CGT AGG AGT			[43]
nonEUB	negative control	-	ACT CCT ACG GGA GGC AGC			[37]
**Helper probes/competitor**						
Betcomp	Competitor for Bet42a		GCC TTC CCA CAT CGT TT			[37]
Bac1157comp	Competitor for Bac1157		CCATTGGTATCAATTCGCAGC			
Bmal16S95comp	Competitor for Bmal16S95		CGT TCG CCA CTC GCC A			[39]
Bru-996comp	Competitor for Bru-996		CCA CTA ACC GCG ATC GGG ATG			[26]
Bwall1448comp	Competitor for Bwall1448		CAA CCA TCC GCC AGG CCT			[25]
Coxb0187comp1	Competitor for Coxb0187		ATC CCC TGC TTT GCT CCA A			
Coxb187comp2	Competitor for Coxb0187		ATC CCA CGC TTT GCT CCA A			
Gamcomp	Competitor for Gam42a		GCC TTC CCA CTT CGT TT			[37]
HVchol1405R	Helper probe for Vchol1405		CCC ACC TAG CCT TCT CCG TC			
NeMe183comp	Competitor for NeMe183		CCT GCT TTC CCT CTC AAG A			[15]
Ypseudo1531LNA	Competitor for Ypest1531LNA		AAC TGC TTC TGC ACC GT+A GTG			

* indicates probes used at 30% formamide or 3.5 M urea. All other probes were used at 35% formamide or 5 M urea, except for EUB and nonEUB, which were used as controls at either 30% formamide, 35% formamide, 3.5 M urea or 5 M urea. + indicates the incorporation of a locked nucleic acid at the respective site.

^§^probes designed in this study were evaluated for coverage and specificity using the TestProbe-tool (https://www.arb-silva.de/search/testprobe) and matched against SSU138 and LSU132 respectively [34]. Coverage values are related to the clinical relevant target organisms. Specificity is indicated as number of full–match-hits to nontarget organisms in the Silva-database.

### Fluorescence in situ hybridization and clone-FISH

FISH was performed as previously described [36]. Briefly, 2 μl of fixed samples were spotted on slides, allowed to dry and dehydrated in an ascending ethanol series (50%, 80% and 96% ethanol for 3 minutes each). 10 μl hybridization buffer (0.9 M NaCl, 20 mM TrisHCl (pH 8.0), 0.01% SDS, 0–50% formamide (concentration depending on the probe used)) were mixed with the respective probes, applied to the microscope wells, and slides were incubated at 46°C in a humid chamber in the dark for one hour. Slides were washed for 10 min in washing buffer (0.009 M– 0.45 M NaCl (adjusted according to the formamide concentration of the hybridization buffer), 20 mM TrisHCl (pH 8.0), 5 mM EDTA (pH 8.0)), dipped in ice-cold water and dried immediately. Gram-positive bacteria were permeabilized with 15 mg/ml lysozyme (Cat.Nr. 62970, Fluka) for 10 minutes at 37°C prior to hybridization. For the evaluation of the probe Rick2287 clone-FISH was performed [44]. Briefly, full-length 23S ribosomal RNA genes of *Rickettsia bellii* and *Orientia tsutsugamushi* (DNA kindly provided by Dr. Christian Keller, Philipps-University, Marburg) were amplified with the primers Rick74f (5’-AGC TTC GGG GAG YTG CGA) and Rick2713r (5’-CGT ACT TAG CTA CCC AGCT A), cloned into the pCR II-TOPO vector (Invitrogen, Carlsbad, USA) and transformed into *E*. *coli* BL21 (DE3) competent cells (Invitrogen, Carlsbad, USA). Transcription was induced in the presence of 270 μg/ml Chloramphenicol and 100 μg/ml Kanamycin by the addition of 1 mM isopropyl-beta-D-thiogalactopyranoside to the medium. Cells were harvested five hours after induction and fixed with formaldehyde as described above. Untransformed *E*. *coli* cells and non-induced cells harboring the plasmid were used as additional controls in the clone-FISH experiments.

Images for the evaluation of formamide series were acquired using a confocal laser scanning microscope (LSM 710, Zeiss) and analyzed using the daime software [45].

### Fluorescence in situ hybridization with urea for field applications

Fixation and dehydration of samples was done as described above for FISH with formamide-containing hybridization buffer. Urea concentrations for the set of probes used in this study were optimized by evaluating the signal intensities of target and non-target organisms at different urea concentrations. The final urea-based hybridization buffers used in all experiments contained 0.45 M NaCl, 20 mM TrisHCl (pH 8.0), 5 M urea and 0.01% SDS for probes routinely used with 35% formamide or 3.5 M urea for probes used with 30% formamide. The corresponding washing buffers contained 0.3 M NaCl, 20 M TrisHCl (pH 8.0), 5 M urea and 5 mM EDTA (pH 8.0) or 3.5 M urea, respectively. In order to compare signal intensities, hybridizations with formamide and urea were performed in parallel, images were acquired with identical settings and analyzed with the daime software [18].

For lyophilization, hybridization and washing buffer containing urea as well as mixtures of probes in hybridization buffer were prepared, aliquots of 500 μl of buffers and 26 μl of probe mixtures were lyophilized and stored at room temperature. Hybridizations with lyophilized and fresh reagents were performed in parallel directly after lyophilization and after four weeks of storage time, followed by image acquisition with identical settings and comparison of signal intensities with daime. Hybridizations in the field were carried out in an Inca mini microtiter plate incubator with urea-based, lyophilized reagents as described above. Slides were observed using a CyScope II fluorescence microscope (Sysmex Partec, Goerlitz, Germany) and images were acquired through the eyepiece of the microscope with a conventional smartphone (Galaxy S5, Samsung).

### Development of an algorithm for the identification of biological agents and its application to surrogate matrices

In order to allow for a fast identification of the thirteen target organisms used in our panel, up to six double-labeled probes with the respective competitor and helper probes were combined in a single hybridization experiment [46] and applied to clinical samples or pure cultures spiked into different sample matrices. In all experiments, controls with the EUB probe, targeting all bacteria, and the nonEUB probe, acting as a negative control to monitor unspecific binding of probes, were included. For clinical samples, an additional positive control consisting of a mixture of known bacteria was included on an extra slide to control for successful binding of probes. FISH was performed as described for pure cultures. Samples were additionally stained with 4’, 6-diamidino-2-phenylindole (DAPI) to detect all microorganisms and eukaryotic cell nuclei present in the samples. If the presence of Gram-positive bacteria was suspected, samples were analyzed both with and without lysozyme treatment for FISH. Gram-positive bacteria were permeabilized with 15 mg/ml lysozyme (Cat.Nr. 62970, Fluka) for 10 minutes at 46°C prior to hybridization.

Powder samples were produced by mixing radiation-inactivated and lyophilized *Y*. *pestis* with an inorganic carrier matrix. A small amount of the powder-bacteria mixture was added to 500 μl PBS and vortexed. Larger particles were allowed to settle for several minutes and the supernatant was used for FISH as described above. For sampling of surfaces, tapes of different manufacturers were evaluated regarding their suitability for FISH with regard to autofluorescence, handling and stickiness. For skin sampling, pig trotters were purchased at a local supermarket. *E*. *coli* Nissle was first grown in LB medium, and the pork skin was inoculated by streaking a swab drenched with *E*. *coli* over its surface and incubated overnight at room temperature. Samples were taken from the skin surface using tape (tesafilm kristall-klar) as previously described [47]. Bacteria were fixed on tape with 4% formaldehyde in PBS for an hour and all further hybridization and washing steps were performed on tape [48]. Sporulation of *B*. *anthracis* was induced as previously described [49]. Vegetative cells were eliminated by heating the samples to 65°C for 30 minutes, followed by fixation in 10% formaldehyde for two hours. The extent of sporulation in the samples and the structural characteristics of spores were checked by light and scanning electron microscopy (S1 Fig). For the determination of spore numbers, spore suspensions were diluted in PBS and counted using disposable plastic counting chambers that were sealed with nail polish. For FISH, fixed spores were applied to glass slides and the spore coat was permeabilized for FISH-probes by the following incubations: 15 minutes at 65°C in a mixture of 7 M urea, 1% SDS, 10 mM TrisHCl and 50 mM DTT followed by a washing step; 30 min at 37° with proteinase K (Qiagen, Hilden, Germany) in 2 M urea followed by a washing step; 15 minutes at 37°C in a mixture of lysozyme (15 mg/ml) and mutanolysin (0.1 mg/ml) in PBS (pH 7.2); one hour at 37°C in 1% SDS and proteinase K in PBS (pH 6.5). After this treatment, FISH was performed as described above.

## Results

### Urea as a non-toxic substitute for formamide in FISH

**Fig 1:**
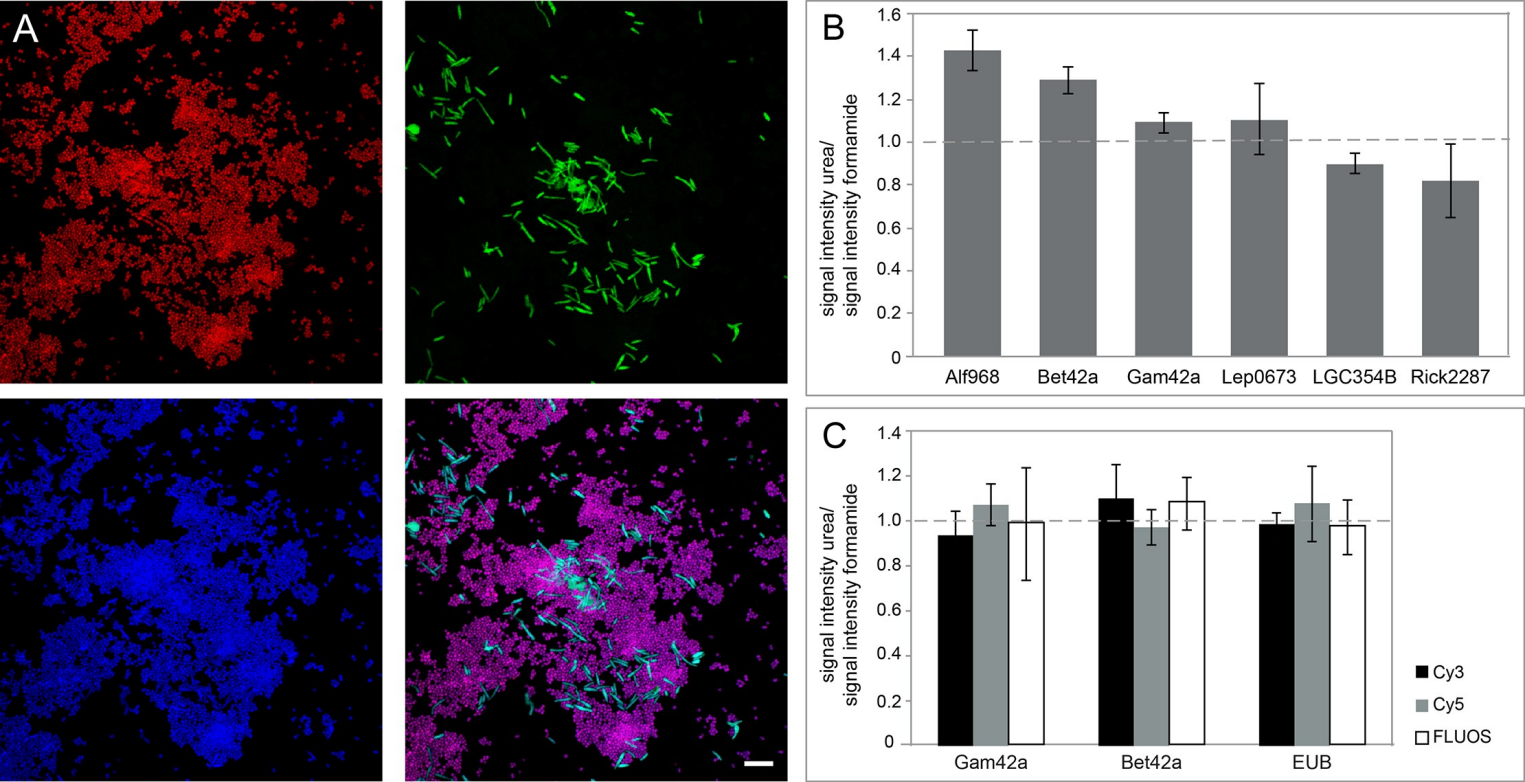
FISH with urea-based buffers is specific and similar in signal intensity to hybridizations with formamide. **(**A) Specific detection of *E*. *coli* by the probe Gam42a (red), *N*. *meningitidis* by the probe Bet42a (green), and both organisms by the EUB probe (blue) in hybridizations with urea. In the lower right corner, an overlay of the three images is shown. Absence of yellow color indicates that there was no unspecific double hybridization of Cy3 (red)—and Fluos (green)—labelled probes. Bar 10 μm. (B) Ratios of signal intensities after hybridizations with urea and formamide for six different probes. Ratios above 1 indicate higher signal intensities in hybridizations with urea-based reagents; ratios below 1 indicate higher signal intensities in hybridizations with formamide. Ratios are based on at least three replicates and the standard deviation is shown for each ratio. (C) Ratios for the signal intensities after hybridization with urea and formamide-based reagents with different fluorophores. Ratios are based on at least three replicates (min. 670 cells/replicate) and the standard deviation is shown for each ratio.

In order to establish a field-applicable FISH protocol that can be used in the absence of a fume hood, we searched for a non-toxic alternative to formamide. Urea has been used to adjust the stringency of hybridizations for the detection of *Staphylococcus aureus* in a previous study [31]. We reevaluated this protocol with the probes Gam42a (targeting most *Gammaproteobacteria*) and Bet42a (targeting most *Betaproteobacteria*) and the respective competitor probes with *Escherichia coli* and *Neisseria meningitidis*. These two probes target the same binding site of the 23S rRNA and feature only one mismatch to each other, but binding is specific under stringent conditions [37]. With the previously published urea-based hybridization protocol [31] intense signals with both probes were observed for both organisms even in the presence of competitor probes, indicating unspecific binding. Therefore, we adjusted the salt- and urea-concentrations of the hybridization- and washing buffers in subsequent experiments until signals were obtained only for the respective target organism, but no longer for the non-target organism (Fig 1A and S2 Table). No signals were observed when the nonsense probe nonEUB was used as a control.

Subsequently, we expanded the urea-based hybridizations to a set of bacterial pathogens including some that have been categorized as potential biological warfare agents (Table 1). Where probes had already been published for the respective organism, we reevaluated the specificity of these probes using the SILVA database (SILVA SSU138/LSU 132) [34] and BLAST. Newly designed probes were first evaluated using the standard formamide-based hybridization buffer and were subsequently tested with the urea-based reagents. All sixteen probes were specific for the respective target organisms in formamide-based hybridizations. Due to the presence of sequence variabilities in the multiple rRNA operons of *Yersinia* strains, Ypest1531LNA bound to the rRNA of both *Y*. *pestis* and *Y*. *pseudotuberculosis*. However, discrimination of the two organisms was possible using a fluorescently labeled competitor containing a locked nucleic acid (LNA) targeting *Y*. *pseudotuberculosis* that specifically detected *Y*. *pseudotuberculosis*, but not *Y*. *pestis* (S2 Fig). The incorporation of LNA-bases in FISH-probes has also been reported to increase the stringency of FISH-probes in other studies [27, 50, 51]. Urea-based hybridizations exhibited specificity identical to formamide-based FISH.

To further test whether changes in buffer composition have an influence on signal intensity, we compared the mean signal intensities for six probes targeting different phylogenetic groups after hybridizations with formamide and urea, respectively. For four probes, signal intensities were similar under both conditions (n = 3; min. 180 cells/replicate) (Fig 1B). Rick2287 (targeting *Rickettsia* spp.) gave higher signal intensities in hybridizations with formamide, while Alf968 (targeting part of the *Alphaproteobacteria*) gave brighter signals with urea (Fig 1B). Urea-to-formamide signal intensity ratios were dye-independent as no change was observed when the probes Bet42a, Gam42a and EUB338-I were labeled with either Cy3, Cy5 or FLUOS in hybridizations, respectively (Fig 1C).

### Field-applicable FISH

**Fig 2:**
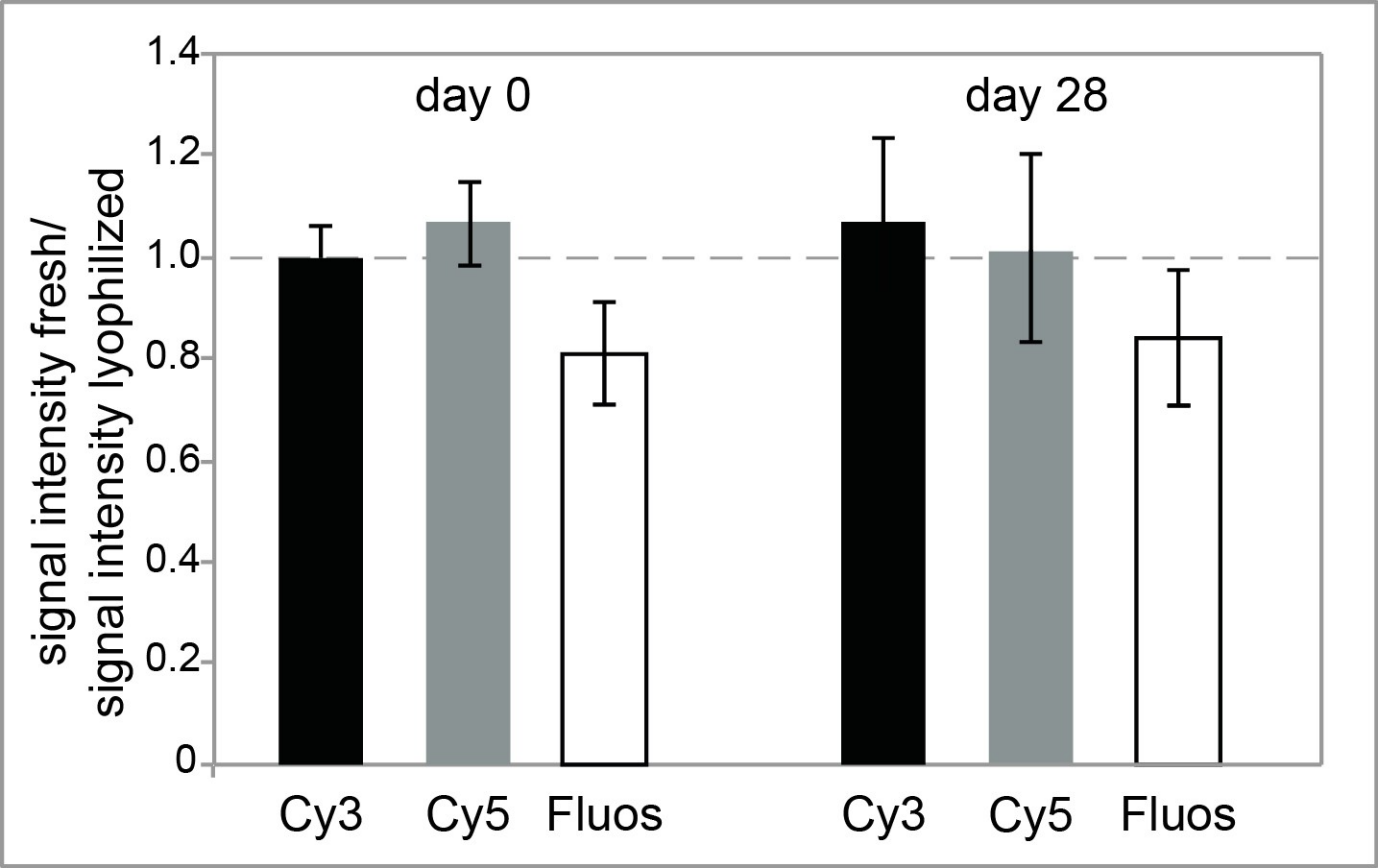
Influence of lyophilization and prolonged storage on the probe signal intensity in urea-based hybridizations. The Cy3-labeled Gam42a, Cy5-labeled Bet42a and FLUOS-labeled EUB probes were lyophilized and used in hybridizations either directly after lyophilization or after storage at room temperature for 28 days. Signal intensities were compared to signal intensities of freshly prepared reagents without lyophilization. A ratio of 1 means no change, while ratios below 1 indicate negative influence of lyophilization or storage. *E*. *coli* was used as target organism for Gam42a, *N*. *meningitidis* for Bet42a and both organisms as target for the EUB probe. The means and the standard deviation based on at least three replicates are shown.

For application in the field, any technique should require only light-weighted equipment, take up as little space as possible, and use reagents that can be transported cold-chain independently [29]. Accordingly, the equipment used for NOTIFy-FISH (S3 Table) was optimized concerning weight and size, making it easy to transport. To further facilitate cold-chain independent transportation, we tested lyophilization of urea-based hybridization and washing buffers and probes and compared them to freshly prepared reagents. Lyophilization of buffers and probes did not result in a change in the signal intensity for probes labeled with the cyanine dyes Cy3 and Cy5 and only in a slight decrease for FLUOS-labeled probes (Fig 2). Lyophilization also did not cause unspecific binding of nonsense probes or reduced probe specificity. Storage for up to four weeks at room temperature did not change these results (Fig 2) FISH performed using this reduced equipment and lyophilized reagents resulted in reproducible bright and specific signals (n = 3).

**Fig 3:**
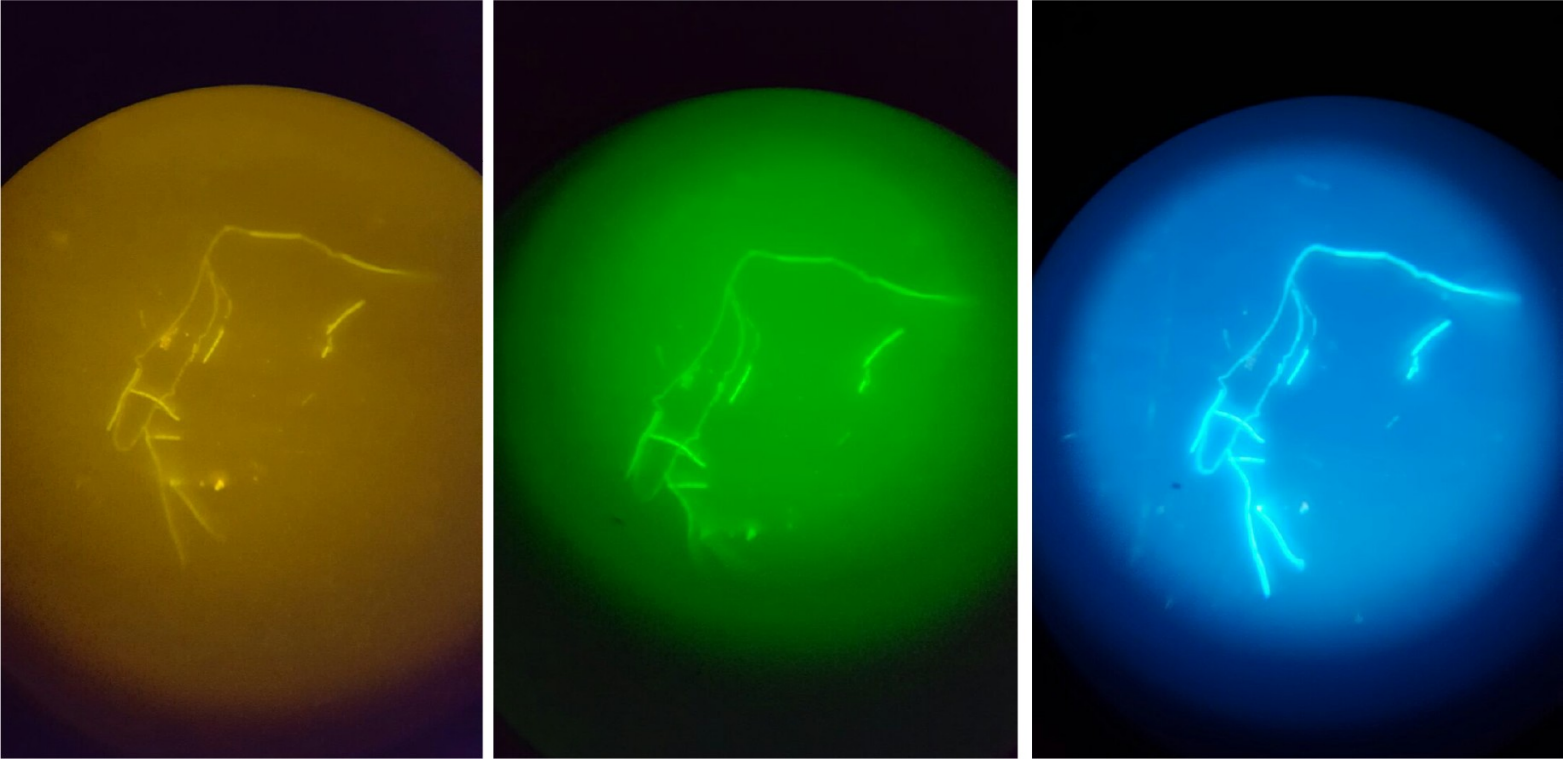
Detection of *Bacillus anthracis* by NotiFy-FISH under field conditions. Signals for *Bacillus anthracis* detected by the probes Bac1157 (left) and EUB (middle) after hybridization with urea-based lyophilized reagents and DAPI staining (right) were recorded with a conventional smartphone through the lens of a portable, battery-operated, fluorescence LED microscope.

Signals could be observed with a portable, battery-operated, fluorescence LED microscope, were recorded using a smartphone camera (Fig 3) and could be clearly distinguished from negative controls.

### A FISH-based algorithm for the identification of biological agents

**Fig 4:**
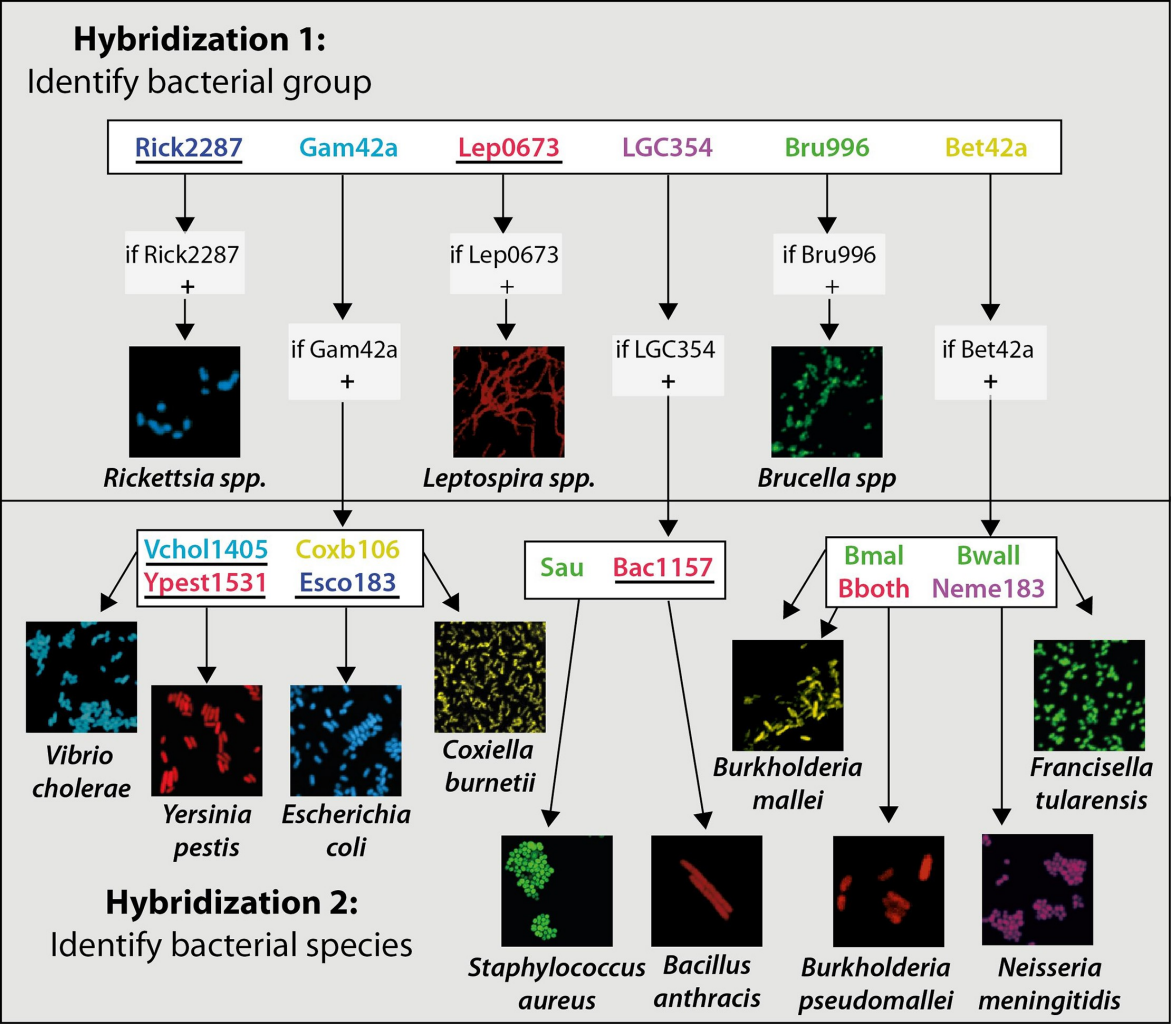
Two-step workflow for the identification of thirteen different organisms by DOPE-FISH. Probes that were designed and evaluated in this study are underlined in black. Note that *B*. *mallei* is detected by the probes Bmal and Bboth, while *B*. *pseudomallei* is detected by Bboth only. *F*. *tularensis* belongs to the *Gammaproteobacteria*, but is detected by the probe Bet42a. Bacterial FISH images are drawn to scale and represent 10 μm squares each.

To allow for a fast identification of the thirteen selected target organisms in the presence of more sophisticated equipment (e.g. in a reach-back laboratory) we set up a two-step diagnostic algorithm combining up to six double-labeled probes per hybridization. The first hybridization allows for the identification of *Rickettsia* spp., *Brucella* spp. and *Leptospira* spp. and an assignment to a bacterial group for all other target organisms. In the second hybridization, a mixture of species-specific probes is used to identify the target organisms within the respective group (Fig 4).

**Fig 5:**
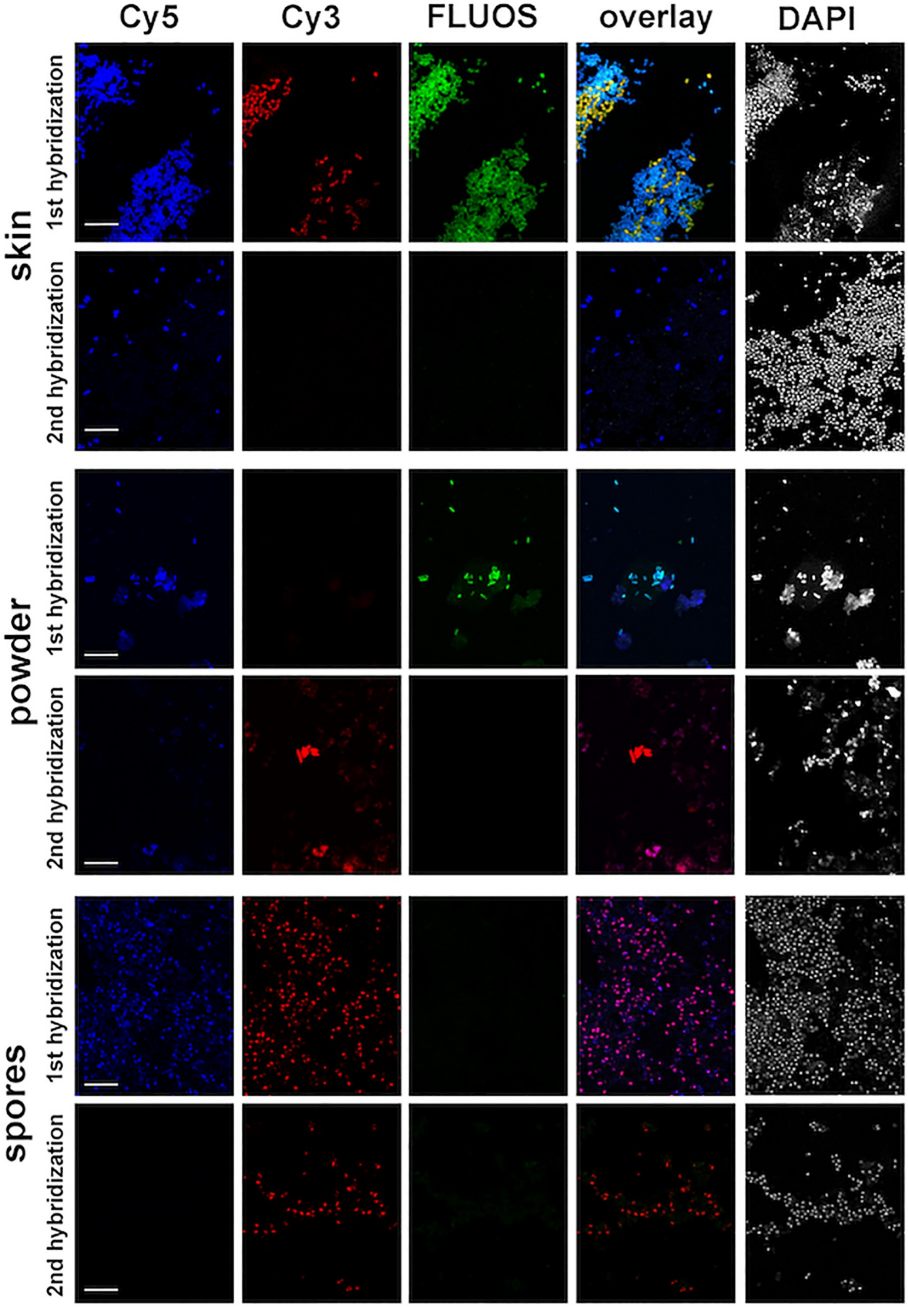
Identification of biological agents on skin surface after sampling by tape, in powder samples and in a spore suspension using the NotiFy-algorithm. *E*. *coli* was detected on porcine skin in the first hybridization by the Gam42a probe (labeled in Cy5 and FLUOS, turquoise color in the overlay) and by the probe Esco864 (labeled in Cy5) in the second hybridization. Additional bacteria were detected in the first hybridization by the Bet42a probe (labeled in Cy3 and FLUOS, yellow color in the overlay) and DAPI or by DAPI alone, but could not be identified by the algorithm. DAPI-signals for bacteria other than *E*. *coli* were also observed in the second hybridization. In powder samples, *Y*. *pestis* was identified by the Gam42a probe (labeled in Cy5 and FLUOS, turquoise color in the overlay) in the first hybridization and by the probe Ypest1531LNA (labeled in Cy3, red color in the overlay) in the second hybridization. Autofluorescent powder material can be observed in all three channels. *B*. *anthracis* spores were detected after pretreatment to break the spore coat with the probe LGC354B (labeled in Cy5 and Cy3, violet color in the overlay) in the first and with the Bac1157 probe (labeled in Cy3) in the second hybridization. Not all spores were accessible to FISH-probes after the pretreatment, resulting in only DAPI signals but no FISH signals for some of the spores. Bars 10 μm.

The algorithm was first tested with pure cultures of target and non-target organisms; probe binding was specific for all target organisms. We then expanded testing to sample matrices, which are likely to be encountered when dealing with suspected bio-terroristic samples. Organisms were successfully identified on skin surfaces, in powder samples and in spore preparations using formamide- as well as urea-based hybridizations (Fig 5).

Porcine skin intentionally contaminated with *E*. *coli* was used as a model for cutaneous exposure to bacteria and sampled with tape. Bright and clearly distinguishable signals with the Gam42a probe and the *E*. *coli* specific probe Esco864 were observed after hybridizations performed on tape (Fig 5). In addition to *E*. *coli*, a high number of *Betaproteobacteria* and other bacteria were detected, representing the autochthonous skin microbiome, but could not be identified with the set of probes used in this study. Detection of bacteria in powder samples was difficult as this typical inorganic carrier matrix showed high autofluorescence. However, even in such a difficult sample matrix the identification of *Yersinia pestis* with probe Gam42a and the species-specific probe Ypest1531LNA could be achieved (Fig 5). Furthermore, rapid detection of *Bacillus anthracis* spores is of particular importance for biodefense applications. Several protocols for the identification of *Bacillus* spp. spores by FISH have been previously published [23, 52–54], but only yielded signals for up to 10% of *B*. *anthracis* spores in our hands. Most of these studies used other *Bacillus* species as a surrogate for *B*. *anthracis*. However, the structure of the elaborate spore coat of bacilli shows variations in different species [55]. In addition, the use of different sporulation conditions regarding media composition and time might also influence the spore coat structure [23]. In combination, this could lead to inadequate permeabilization of cells and low detection efficiencies. We therefore modified the existing protocols, which finally resulted in detection rates of up to approximately 70% for *B*. *anthracis* spores (Fig 5). Not all spores stained by DAPI also gave corresponding FISH signals. To exclude the presence of damaged or dead spores after the purification process, fresh spore preparations were quantified and plated before fixation. Nearly 100% of the spores were found to form colonies on nutrient agar plates, showing germination of the spores and ruling out the presence of damaged spores. Small differences in the coat composition between different subpopulations might lead to different sensitivities to lysozyme and proteinase K, possibly explaining our inability to detect all spores by FISH.

### Application of NOTIFy-FISH to clinical samples

**Fig 6:**
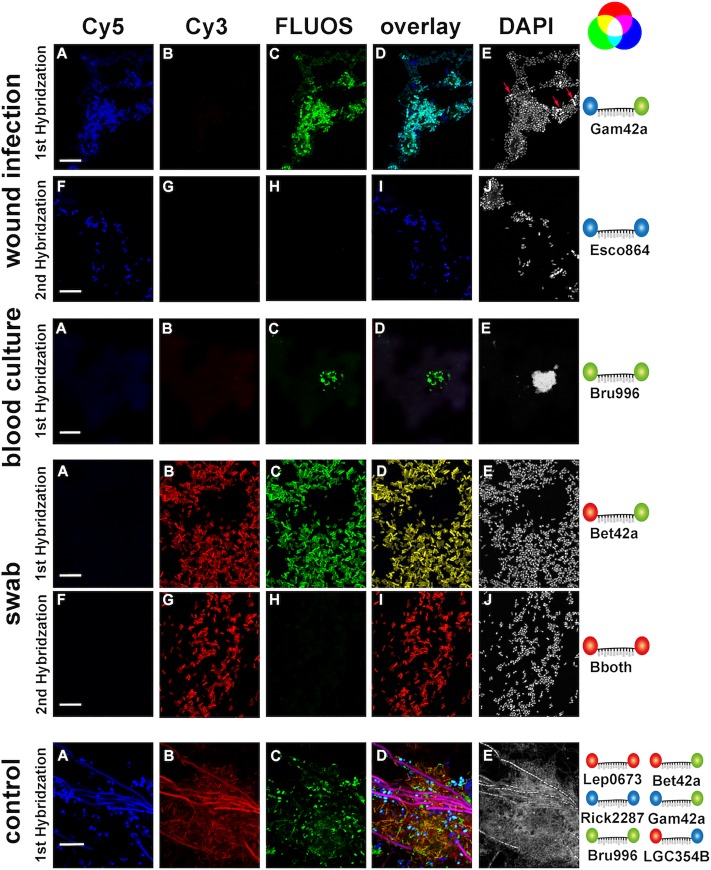
Application of the diagnostic algorithm for the identification of bacteria in clinical samples. *E*. *coli* was identified in an infected skin lesion, *Brucella* spp.in a blood culture and *Burkholderia pseudomallei* in a swab of an infected patient. In the infected skin lesion, bacteria that were only detect by DAPI, but not by any of the other probes used were present (arrows). Efficient probe binding was controlled with a positive control containing a mixture of known bacteria (control). Shown are images acquired after hybridization with formamide-based (wound infection and control) or urea-based reagents (blood culture and swab). *E*. *coli* was detected by the probe Gam42a (labeled in Cy5 and FLUOS, turquoise color in the overlay) in the first hybridization and identified by the probe Esco864 (labeled in Cy5) in the second hybridization. *Brucella* was identified by Bru996 (labeled in FLUOS) in the first hybridization. *B*. *pseudomallei* was detected by the probe Bet42a (labeled in Cy3 and FLUOS, yellow color in the overlay) in the first hybridization and identified with the probe Bboth (labeled in Cy3) in the second hybridization. In the control overlay, specific binding of the probes used in the first hybridization is shown by detection of *B*. *anthracis* in pink, *Y*. *pestis* in turquoise, *B*. *suis* in green, *L*. *borgpetersenii* in red, *B*. *pseudomallei* in yellow and *R*. *slovaca* in blue. The probes and fluorophores resulting in signals in the respective sample are indicated on the far right under a representation of the additive mixing of colors. Bars 10 μm.

All the organisms targeted by the NOTIFy algorithm are not only of interest due to their potential use as biological agents, but can also cause naturally occurring, serious and often life-threatening infections in humans. To test whether the developed algorithm is also suitable for the identification of these bacteria in clinical samples, we applied it to 20 samples from 15 patients received in a medical microbiology lab for diagnostics of the target agents of this study (S4 Table). Analysis was performed using formamide- and urea-based hybridization buffers. For all hybridizations, controls with nonsense probes and positive controls consisting of a mixture of known bacterial pure cultures were included (Fig 6).

NOTIFy-FISH results were compared to the standard diagnostic procedures (qPCR assays and bacterial cultures) (S4 Table). In six of the 20 samples the suspected causative bacteria could be verified by cultivation. Of these six cultivation positive samples, all but one also showed positive results for the same bacterium with FISH. *Brucella* spp. was identified in a set of blood cultures from a patient (one aerobic and one anaerobic bottle) by bright signals with the probe Bru996 that were clearly distinguishable from the slight autofluorescent background of blood cells (Fig 6). In another set of clinical samples, *Burkholderia pseudomallei* was identified by application of the FISH algorithm (Fig 6). Although results obtained by cultivation and FISH largely overlapped, identification by FISH proved to be up to 24 hours faster than culturing attempts in accordance with previous studies [15]. For some samples, microscopy gave additional insights. In the swab from a skin lesion of a patient with suspected tularemia, three different bacterial morphotypes were clearly distinguishable by DAPI staining. One of these could be identified as *E*. *coli* by FISH, while the other two morphotypes represented a *Gammaproteobacterium* and a Gram-positive bacterium which could not be further differentiated by our set of probes (Fig 6). Cultivation of the skin lesion swab samples on agar plates followed by MALDI-TOF spectrometry confirmed the growth of *Streptococcus pyogenes*, *Escherichia coli* and *Acinetobacter junii*. The suspected pathogen *F*. *tularensis* was not detected in this sample by any method. For one sample where *Brucella* spp. and one sample where *F*. *tularensis* were detected by qPCR, both FISH and culturing attempts were unsuccessful. This suggests that bacterial concentrations in these samples were either low or nucleic acid material of dead bacteria was detected by qPCR, as PCR would also yield positive results for DNA remnants of lysed cells.

## Discussion

A prerequisite for the reliable identification of biological agents is the confirmation by several independent methods [1]. To broaden the spectrum of available techniques, we established a set of FISH-probes and a protocol applicable for the microscopy-based identification of biological agents in the field. The NOTIFy approach developed here is fast, non-toxic and requires only limited equipment. This makes it also an interesting option for other field studies or studies where non-toxic conditions are required. Two independent studies using urea-based FISH reported much higher signal intensities for hybridizations with urea compared to formamide [31, 32]. For the set of probes used in the current study, the signal intensity after hybridizations with urea-based reagents varied for each of the probes in comparison to formamide (Fig 1). This might be due to the limited number of probes tested in the previous studies (n = 3) and/or the use of different salt- and urea concentrations in the buffers compared to this study. The use of the protocol published by Lawson [31] also resulted in much higher signal intensities when tested for our set of probes, but binding of the probes was unspecific. In contrast to this, hybridizations performed with the optimized urea-based buffers developed in this study showed the same specificity as formamide-based FISH for all probes in all sample matrices tested. However, for some probes or organisms the optimization or design of new probes might be necessary, because currently available probes have been optimized for formamide-based FISH.

As some of the bacteria tested in this study are highly pathogenic to humans, we used paraformaldehyde for fixation in all experiments in order to reliably ensure inactivation of biological material. For Gram-positive bacteria, especially in the late growth stage, ethanol-fixation was shown to give superior signal intensities [56]. However, as treatment with ethanol does not guarantee an inactivation of *B*. *anthracis* spores [57], we did not evaluate ethanol as an alternative fixation strategy in combination with urea.

Powder samples suspected to contain spores of *B*. *anthracis* are regarded as a typical matrix in the context of biodefense scenarios. Although spores are difficult to penetrate for FISH-probes due to their rigid spore coat, they pose excellent targets for FISH, as their rRNA content is comparable to vegetative cells in the log phase [58]. Papers for the detection of *Bacillus* spp. and *B*. *anthracis* spores by FISH have been published [23, 52–54], but did not show high detection efficiencies for *B*. *anthracis* spores in our hands. This might be caused by the use of closely related, but different species like *B*. *cereus* in these studies [52–54]. Differences in the structure of the spore coat might hinder the successful transfer of protocols from one bacillus species to the other [55].

One of the drawbacks of FISH is its rather low sensitivity as compared to qPCR [15, 59], although the reported detection limits for FISH vary widely even under similar conditions [7]. However, FISH was shown to perform better than most biosensors for the detection of *B*. *anthracis* spores from air [23]. Another recent study showed that in minced meat, after a short enrichment step, one colony forming unit/gram of *Y*. *enterocolitica* was detectable by FISH, making it more sensitive than currently used ISO methods in food microbiology [27].

FISH has certain advantages over other methods. It is an extremely robust method and less prone to matrix dependent inhibition compared to PCR based methods. Furthermore, the presence of bacteria other than the organism of interest is easily detected as shown by the application of the NOTIFy algorithm in combination with nucleic acid staining by DAPI (Fig 5, skin and Fig 6, wound infection). This can be of high importance if more than one bacterial pathogen is present or in the case of mixed infections in clinical applications [21, 60]. Similar results cannot be achieved by the use of specific primers or selective growth media. In addition, FISH can provide information on the abundance and viability of the targeted bacterium in comparison to other bacteria in the sample as well as on their localization within the sample. In the clinical samples we analyzed, detection of bacteria by FISH always coincided with successful cultivation, but qPCR gave additionally positive results for some of the samples that were negative by FISH. This can be partly attributed to the higher sensitivity of qPCR, but also to the fact that DNA remains detectable even in the absence of intact cells [61, 62]. This suggests that bacterial numbers in these samples were either very low or bacteria were already dead or inactive due to previous antibiotic treatment [61, 62]. Therefore, FISH combined with qPCR might give valuable information about the actual therapeutic success of an antibiotic therapy in a patient, especially since both methods give much faster results than cultivation. Furthermore, organisms like *Coxiella burnetii*, which are viable but not culturable using standard culture media, can be detected in samples. However, we want to point out that we do not regard FISH as a stand-alone technique, but always suggest a combination with nucleic-acid based amplification methods, MALDI-TOF mass spectrometry or standard culturing techniques. Moreover, we want to stress the importance of adequate positive and negative controls especially in clinical diagnostic applications of FISH. Also, fast fixation of obtained sample material is important to ensure high ribosomal content of bacteria and bright signals in FISH.

The FISH-probe based algorithm demonstrated in this paper could be used as a blueprint to establish diagnostic probe sets for further clinically encountered disease patterns. With the inclusion of double-labeled probes in an algorithm, up to 36 different cell types could be differentiated in two hybridizations, opening a large field of possible applications in clinical settings. Moreover, the non-toxic protocol and lyophilized buffer systems described herein might be of great interest to clinical laboratories.

## Supporting information

S1 Fig Preparation of *Bacillus anthracis* spores. Light electron microscopy (A) and scanning electron microscopy (B and C) show the absence of vegetative cells and the presence of fully matured spores. Bars 10 μm for A and B, and 1 μm for C.
(DOCX)

S2 Fig Evaluation of probes Ypest1531LNA and Ypseudo1531LNA. (DOCX)

S1 Table Control organisms and their growth conditions used in this study. (DOCX)

S2 Table Table with Urea based hybridization- and corresponding washing-buffers. (DOCX)

S3 Table Equipment needed to perform NOTIFy-FISH in the field. (DOCX)

S4 Table Clinical samples analyzed by FISH. Results of qPCR using specific primers for the suspected causal bacterium, cultivation and FISH are shown for each sample. *Identified by MALDI-TOF mass spectrometry.
(DOCX)
