# Supplementary material for: NOTIFy (non-toxic lyophilized field)-FISH for the identification of biological agents by Fluorescence in situ Hybridization
Source: PLoS One. 2020 Mar 6;15(3):e0230057. doi: 10.1371/journal.pone.0230057 (PMC7059943; doi:10.1371/journal.pone.0230057)
Supplement: S1 Fig — Light electron microscopy (A) and scanning electron microscopy (B and C) show the absence of vegetative cells and the presence of fully matured spores. Bars 10 μm for A and B, and 1 μm for C. (DOCX) [file pone.0230057.s001.docx]

**
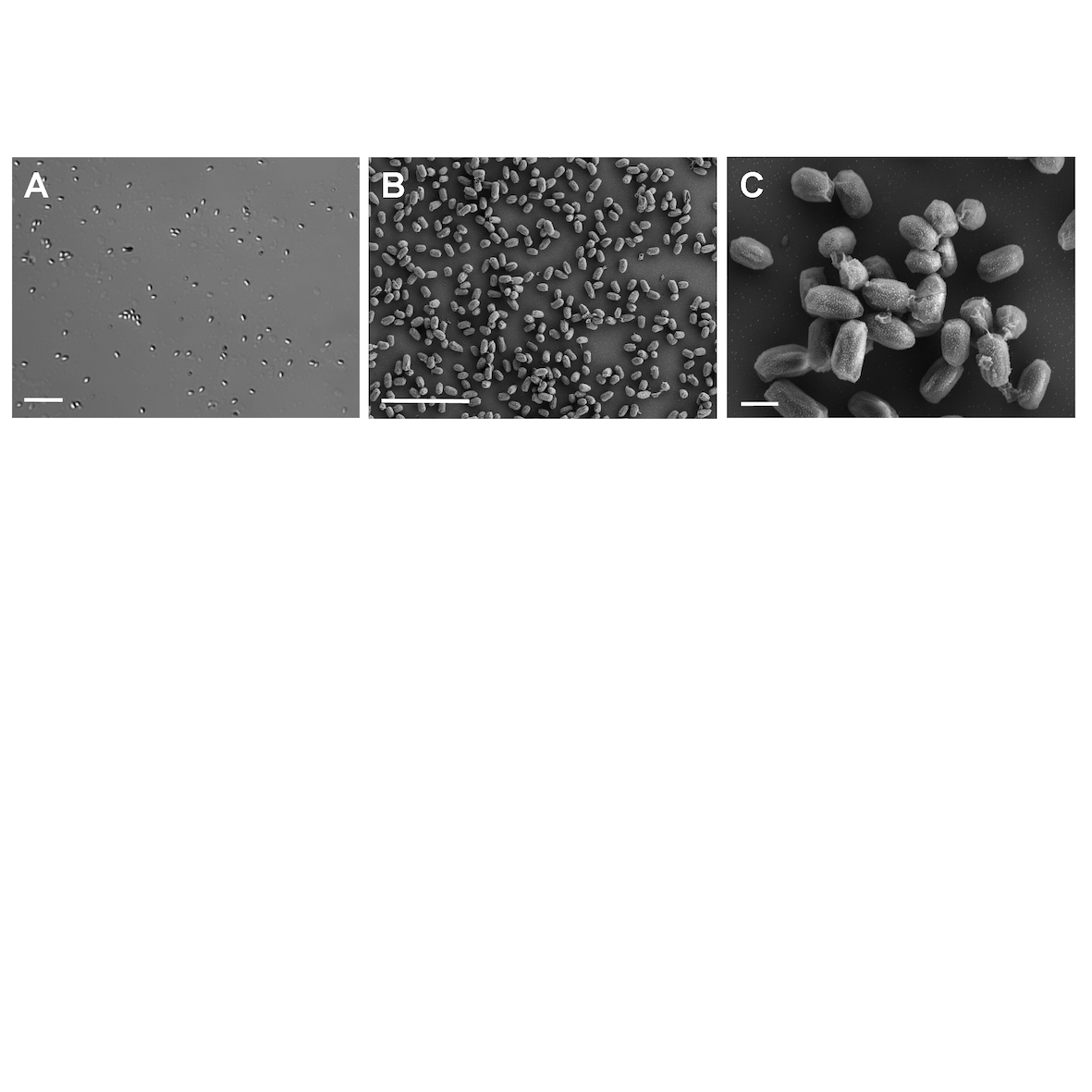
**

**S1 Fig: Preparation of *Bacillus anthracis* spores.** Light electron microscopy (A) and scanning electron microscopy (B and C) show the absence of vegetative cells and the presence of fully matured spores. Bars 10 µm for A and B, and 1 µm for C.
