# Supplementary figures and images for: NOTIFy (non-toxic lyophilized field)-FISH for the identification of biological agents by Fluorescence in situ Hybridization
Source: PLoS One. 2020 Mar 6;15(3):e0230057. doi: 10.1371/journal.pone.0230057 (PMC7059943; doi:10.1371/journal.pone.0230057)

**S2
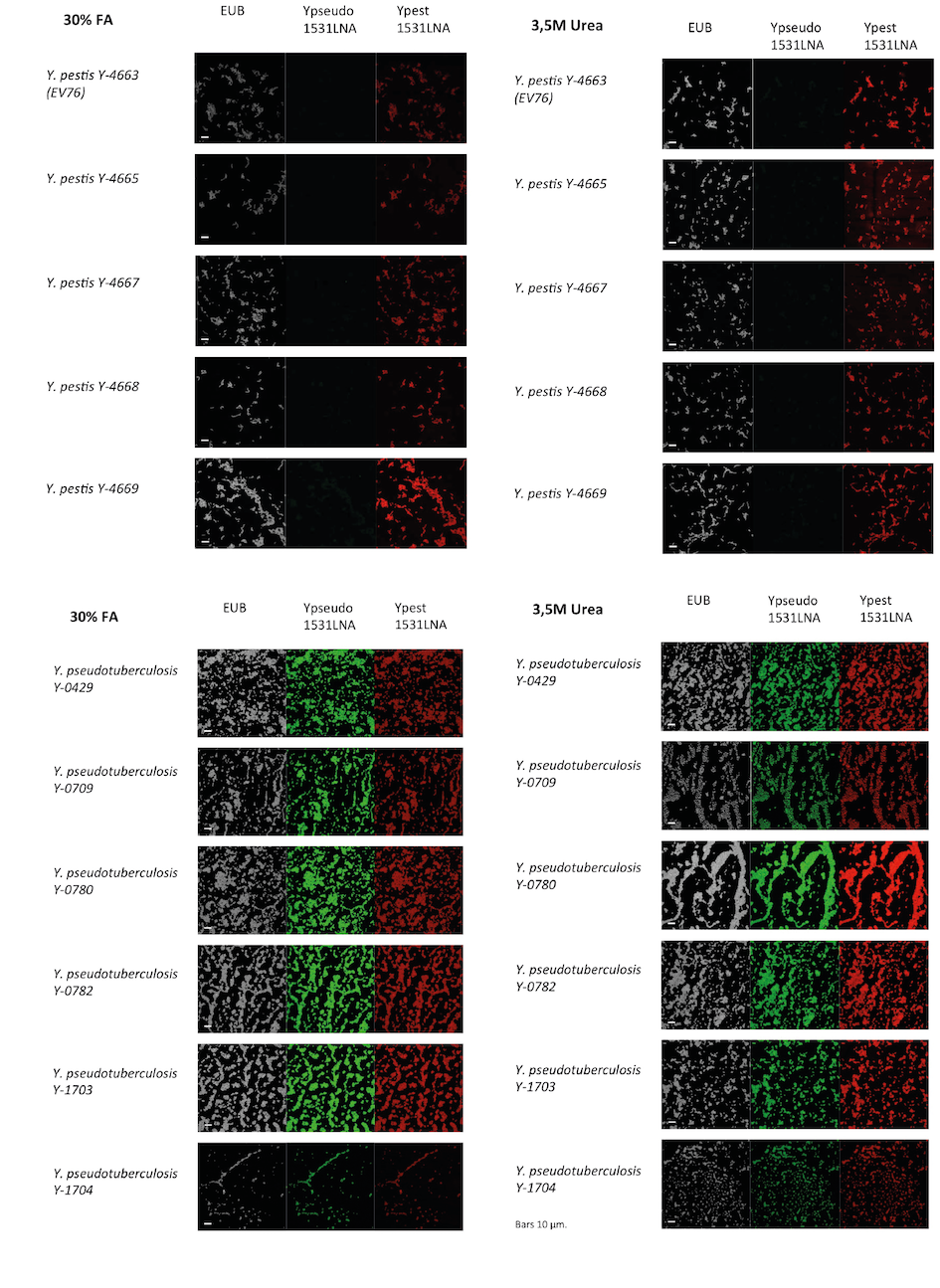
 Fig: Evaluation of probes Ypest1531LNA and Ypseudo1531LNA.**

Bars 10 µm.

Supplement: S2 Fig — (DOCX) [file pone.0230057.s002.docx]
