# Supplementary material for: NOTIFy (non-toxic lyophilized field)-FISH for the identification of biological agents by Fluorescence in situ Hybridization
Source: PLoS One. 2020 Mar 6;15(3):e0230057. doi: 10.1371/journal.pone.0230057 (PMC7059943; doi:10.1371/journal.pone.0230057)
Supplement: S1 Table — (DOCX) [file pone.0230057.s003.docx]

| **Organism** | **Strain** | **Growth temperature** | **Medium** |
| --- | --- | --- | --- |
| *Bacillus thuringensis* | DSM-2046 | 37°C | LB |
| *Bacillus subtilis* | ATCC 6051 | 37°C | LB |
| *Klebsiella oxytoca* | DSM5175 | 37°C | LB |
| *Vibrio cholerae* | ATCC 51394 | 37°C | LB |
| *Burkholderia thailandensis* | E264 | 37°C | LB |
| *Ochrobactrum anthropi* | FO071250 | 37°C | LB |
| *Yersinia pseudotuberculosis* | IMB-141001558 | 28°C | LB |
| *Photobacterium damselae* | DSM-7482 | 37°C | LB |
| *Bacillus cereus* | ATCC 10987 | 37°C | LB |
| *Kurthia gibsonii* | DSM-20636 | 30°C | Corynebacterium medium |
| *Halothiobacillus kellyi* | DSM-13162 | 37°C | Halothiobacillus medium |
| *Yersinia enterocolitica palearctica* O:7/8 | IMB-0022 | 28°C | LB |

**S1 Table: Control organisms and their growth conditions used in this study.**
