# Supplementary material for: NOTIFy (non-toxic lyophilized field)-FISH for the identification of biological agents by Fluorescence in situ Hybridization
Source: PLoS One. 2020 Mar 6;15(3):e0230057. doi: 10.1371/journal.pone.0230057 (PMC7059943; doi:10.1371/journal.pone.0230057)
Supplement: S2 Table — (DOCX) [file pone.0230057.s004.docx]

**S2 Table: table with Urea based hybridization- and corresponding washing-buffers.**
