# Supplementary material for: NOTIFy (non-toxic lyophilized field)-FISH for the identification of biological agents by Fluorescence in situ Hybridization
Source: PLoS One. 2020 Mar 6;15(3):e0230057. doi: 10.1371/journal.pone.0230057 (PMC7059943; doi:10.1371/journal.pone.0230057)
Supplement: S3 Table — (DOCX) [file pone.0230057.s005.docx]

| Microscopy 1 package, 31 kg | | | |
| --- | --- | --- | --- |
| Microscope, Partec CyScope, 2,7 kg | 1 | Cover glass | 100 |
| Polyethylene construction sheeting, 20 m2 | 1 | Disposable plastic sharps container | 1 |
| Purified water, infusion bag, 500 mL | 4 | Glass slides | 100 |
| Staining solution, Gram | 1 | Immersion oil, pack | 1 |
| Staining solution, McFadyean | 1 | Inoculating loop, pack | 3 |
| Storage box for microscope slides | 2 | Laboratory coat, single-use | 8 |
| Tube rack, 50 mL | 2 | Laboratory timer | 1 |
| Tube, cone-shaped, 50 mL | 15 | Pipette Pasteur, single-use | 60 |
| Tweezers, single-use | 2 | Nitrile examination gloves, large | 30 |
| Urine beaker with seal | 2 | Nitrile examination gloves, medium | 20 |
| Waste bags, autoclavable | 5 | Paper wipes, pack | 10 |
| Waste bottle 1 L, Teflon | 1 | Pencil | 1 |
| Inca Personal Microplate Incubator, 920 g | 1 | Glass slides, 10 wells, Teflon coated | 50 |
| Pipette, 100 μL | 1 | PFA solution 10%, 10ml | 1 |
| Pipette, 1000 μL | 1 | PFA solution 4%, 10ml | 1 |
| Filter tips, 1000 μL, box | 1 | Filter tips, 100 μL, box | 2 |
| Plastic double slide shipping container, 25 x 75 mm | 2 |  |  |
| NOTIFy-FISH kit, box with 2ml tubes including lyophilized buffers, probes, positive controls, and lysozyme plus DAPI staining solution, and citifluor mounting medium | | | 2 |

**S3 Table: Equipment needed to perform NOTIFy-FISH in the field.**

Table adapted from [29]
