# Supplementary material for: NOTIFy (non-toxic lyophilized field)-FISH for the identification of biological agents by Fluorescence in situ Hybridization
Source: PLoS One. 2020 Mar 6;15(3):e0230057. doi: 10.1371/journal.pone.0230057 (PMC7059943; doi:10.1371/journal.pone.0230057)
Supplement: S4 Table — Results of qPCR using specific primers for the suspected causal bacterium, cultivation and FISH are shown for each sample. *Identified by MALDI-TOF mass spectrometry. (DOCX) [file pone.0230057.s006.docx]

| **Sample** | **Patient No./**  **Sample No** | **Suspected cause** | **qPCR results** | **Cultivation results** | **Identification by FISH** |
| --- | --- | --- | --- | --- | --- |
| Culture isolate from stool | 1/1 | *B. anthracis* | *B. anthracis* negative, *B. cereus* group positive | *Bacillus* spp from the *B. cereus* group | No |
| Abscess aspiration | 2/1 | *Brucella* spp. | Negative | Negative | No |
| Joint fluid | 3/1 | *Brucella* spp. | Negative | Negative | No |
| Blood culture  (aerobic) | 4/1 | *Brucella* spp. | *Brucella melitensis* | *Brucella melitensis* | Yes |
| Blood culture (anaerobic) | 4/2 | *Brucella* spp. | *Brucella melitensis* | No | Yes |
| Blood culture of ascites sample | 5/1 | *B. pseudomallei* | *B. pseudomallei* | *B. pseudomallei* | Yes |
| Enrichment culture of swab | 5/2 | *B. pseudomallei* | *B. pseudomallei* | *B. pseudomallei* | yes; additional coccoid bacteria detected by DAPI |
| Lymph node | 6/1 | *F. tularensis* | *F. tularensis* holarctica | Streptococcus oralis | No |
| Wound swab from inguinal lesion | 7/1 | *F. tularensis* | Negative | *Streptococcus pyogenes** | Identification of *E. coli*; additional bacteria detected by DAPI |
| Wound swab from inguinal lesion | 7/2 | *F. tularensis* | Negative | *S. pyogenes**, *E. coli**, *Acinetobacter junii** | Identification of *E. coli*; additional bacteria detected by DAPI |
| Wound swab from inguinal lesion | 7/3 | *F. tularensis* | Negative | *S. pyogenes**, *Acinetobacter junii** | Identification of *E. coli*; additional bacteria detected by DAPI |
| Culture isolate | 8/1 | *Brucella* spp*.* | *B. melitensis* | *B. melitensis* | Yes |
| Abscess aspiration | 9/1 | *Brucella* spp*.* | Negative | Negative | No |
| Culture isolate | 10/1 | *Brucella* spp*.* | *B. melitensis* | *B. melitensis* | Yes |
| Abscess aspiration | 11/1 | *Brucella* spp*.* | Negative | Negative | No |
| Bronchial lavage | 12/1 | *B. pseudomallei* | Negative | Negative | No |
| Bronchial lavage | 12/1 | *B. pseudomallei* | Negative | Negative | No |
| Osteochondral material (follow up of antibiotic treated patient) | 13/1 | *Brucella* spp*.* | Negative | Negative | No |
| Biopsy | 14/1 | *F. tularensis* | Negative | Negative | No |
| Lymph node | 15/1 | *Brucella* spp*.* | *B. melitensis* | *B. melitensis* | No |

**S4 Table: Clinical samples analyzed by FISH.** Results of qPCR using specific primers for the suspected causal bacterium, cultivation and FISH are shown for each sample. *Identified by MALDI-TOF mass spectrometry
